# Epidemiology of Breed-Related Mast Cell Tumour Occurrence and Prognostic Significance of Clinical Features in a Defined Population of Dogs in West-Central Italy

**DOI:** 10.3390/vetsci6020053

**Published:** 2019-06-06

**Authors:** Alessio Pierini, George Lubas, Eleonora Gori, Diana Binanti, Francesca Millanta, Veronica Marchetti

**Affiliations:** 1 Department of Veterinary Science, University of Pisa, via Livornese, San Piero a Grado, 56122 Pisa, Italy; george.lubas@unipi.it (G.L.); eleonora.gori@vet.unipi.it (E.G.); francesca.millanta@unipi.it (F.M.); veronica.marchetti@unipi.it (V.M.); 2 AbLab, Veterinary Diagnostic Laboratory, via massa neri, 13, 19038 Sarzana (SP), Italy; diana.binanti@gmail.com

**Keywords:** boxer, canine, metastasis, prognosis, risk factor

## Abstract

Canine mast cell tumours (MCTs) present a wide variety of challenging clinical behaviours in terms of predicting the prognosis and choosing appropriate treatment. This study investigated the frequency, risk, and prognostic factors of MCTs in dogs admitted to a single veterinary teaching hospital (VTH). Breed, age, sex, and sexual status in ninety-eight dogs with MCTs (MCT-group) were compared with a control group of 13,077 dogs (VTH-group) obtained from the VTH clinical database from January 2010 to January 2016. Within the MCT-group, signalment, location, size, mass number, ulceration, histopathological grading, presence of lymph node, or distant metastases were compared with each other and with the outcome. Boxers (OR 7.2), American Pit Bull Terriers (OR 5.4), French Bulldogs (OR 4.4) and Labrador Retrievers (OR 2.6) were overrepresented. The MCT-group was significantly older than the VTH-group (*p* < 0.0001). In comparison with the VTH group, in the MCT-group neutered dogs (OR 2.1) and spayed females (OR 2.3) were predominant compared to intact dogs and intact females, respectively. Ulceration (OR 5.2) and lymph node metastasis (OR 7.1) occurred more frequently in larger MCTs. Both ulceration and MCTs > 3 cm were highly associated with lymph node metastasis (OR 24.8). Recurrence was associated with MCT-related death (OR 10.50, *p* = 0.0040), and the latter was associated with shorter survival times (*p* = 0.0115). Dogs with MCTs > 3 cm (*p* = 0.0040), lymph node metastasis (*p* = 0.0234), or elevated WHO stage (*p* = 0.0158) had shorter survival times. A significantly higher frequency of MCTs was found in specific breeds, and in older and neutered dogs. MCTs > 3 cm and lymph node or distant metastases were associated with shorter survival times.

## 1. Introduction

Mast cell tumours (MCTs) are the most common canine cutaneous tumours, accounting for 16–21% of all skin tumours [1,2]. MCTs may exhibit benign or aggressive clinical behaviour. Some MCTs show a slow growth rate and can be managed and cured by surgical excision alone, whereas others tend to grow quickly, showing early lymph node and distant metastases. These latter MCTs can lead to death, even if treated with a combination of surgery, chemotherapy, or radiation therapy [3,4]. The wide variety of clinical behaviour presented by MCTs makes it challenging for clinicians to provide owners with an accurate prognosis.

To predict the outcomes of dogs with MCTs, many prognostic factors are needed to make treatment decisions. Among the clinical prognostic factors, breed, age, size, and anatomical location of the MCT, single or multiple nodules, tumour ulceration, and lymph node and visceral metastases have been associated with the outcome [5,6,7,8]. Some pure breeds, including Boxer, Boston Terrier, Labrador Retriever, Golden Retriever, Staffordshire Bull Terrier, and Pug, are predisposed to MCTs [6,9]. In addition, MCTs in Pugs and Boxers have been reported to be associated with benign behaviour [1,6,10].

Despite clinical factors, the histopathological grade of MCTs and other pathological markers are commonly used as prognostic factors. The 3-tier Patnaik grading system (G*n*P) is used to predict the outcome for G1P and G3P, but not for G2P MCT [11]. However, G2P MCTs occur frequently, accounting for over 40% of MCTs [5,11,12], resulting in challenging decision-making. Moreover, the Patnaik grading system does not take into account subcutaneous MCTs, which are classified as a separate entity [13]. 

A 2-tier Kiupel grading system (high- or low-grade Kiupel [HGK or LGK]) together with G*n*P provides a better prediction of the outcome for G2P-HGK [5,14]. However, G2P-LGK MCTs have shown a 17% metastatic rate and their behaviour remains difficult to predict [14]. 

The aims of this study were to: (1) investigate the frequency of MCTs in an Italian veterinary teaching hospital (VTH) caseload in West-Central Italy; (2) assess whether breed, sex and sexual status are associated with the frequency of MCTs; (3) evaluate the association between various clinical variables in MCTs; and (4) describe the survival analysis of dogs affected by MCTs.

## 2. Materials and Methods 

The medical record database at our veterinary teaching hospital was searched to identify clinical records of dogs with MCTs between January 2010 and January 2016. Dogs with a cytological or histological diagnosis of cutaneous MCTs, or with cytologically confirmed metastasis of cutaneous MCTs not histologically investigated, were enrolled in the study. Dogs with cytologically suspected MCTs without a histological confirmation or follow-up confirming diagnosis were excluded.

Breed, age, sex, and sexual status in dogs with MCTs were compared with a control group of dogs without MCTs obtained from the clinical database of dogs admitted to our veterinary teaching hospital (VTH group) during the same study period. Each breed and group of breeds were analysed to test the frequency of MCTs. Only breeds with at least 50 dogs in the VTH group were analysed. 

For each dog with an MCT, information regarding signalment, macroscopic tumour features, such as location, size, mass number, and ulceration, histopathological Kiupel and Patnaik grading, histopathological margins, and presence of lymph node or distant metastases were reviewed. For multiple MCTs, the size of the largest nodule and the highest Patnaik and Kiupel grades were used. Tumours were divided into ones that were larger or smaller than 3 cm. Presence or absence of lymph node metastasis was considered as “documented” if a cytological or histopathological report of the regional lymph node was available, and as “presumed” if the lymph node was considered not palpable or undetectable. The presence or absence of distant metastasis was considered only in dogs with a cytological or histological report of liver, spleen, blood, bone marrow, or other organs/tissue far from the primary tumour. Follow-up information was collected via further consultation or phone call with the owners. Local recurrences and locoregional or distant metastases were considered as recurrence. Distant (>10 cm) new MCTs were not considered as local recurrence. The recurrence rate (RR) was calculated. The survival time (ST) was calculated as the time from the day of admission to the day of death due to any cause. Dogs were censored from survival analysis at the last day of available contact. Dogs with unavailable follow-up information were excluded from the survival analysis.

Descriptive statistics were recorded regarding the signalment, body weight, location (extremity, head, trunk and other locations), size, presence of single or multiple MCTs, ulceration, lymph node or distant metastases, WHO staging, Patnaik and Kiupel grade, and margins. 

The extremity location ranged from the elbow or knee to toes. The head location included ears and neck caudally, but not the oral cavity and mucocutaneous junctions. Other MCT locations included the scrotum, perineal, and axillary region, or mucous and mucocutaneous locations. The trunk included all other anatomical regions.

GraphPad Prism version 6.00 (GraphPad Software, La Jolla, CA, USA) was used for statistical analyses. Datasets were tested for normality using the D’Agostino and Pearson omnibus normality test. Values were expressed either as mean and standard deviation in the case of normal distribution, or as median and range with non-normal distributions. Size was analysed both as a continuous variable, when accurate measures (*n* = 73) were available, and as categorical data (< or >3 cm) (*n* = 86). The Fisher’s exact test and the Chi-square test were used to study associations between categorical variables and the Odds Ratio (OR) was calculated. The Kaplan–Meier survival analysis and the logrank test of Cox–Mantel were used to compare the curves within each categorical variable. In all statistical comparisons, *p* < 0.05 was accepted as a statistically significant difference.

## 3. Results

Ninety-eight out of 13,175 dogs (0.77%) had a diagnosis of MCT. Mixed-breed dogs were most common (24.5%), followed by Boxers (23.5%) and Labrador Retrievers (15.3%) (Table 1). Ages ranged from 3.5 months to 15.0 years (median 8.8 years). There were fifty-one females, of which 25 were intact and 26 spayed; and 47 males, of which 43 were intact and 4 neutered. 

Regarding the breeds in the VTH-group, mixed-breed dogs were most common (27.1%), followed by Labrador Retrievers (6.6%) and German Shepherds (5.6%). The median age was 6.22 years (range 4 months–20.4 years). There were 4372 (33.4%) intact females, 1966 (15.1%) spayed females, and 6427 (49.1%) intact males and 312 (2.4%) neutered males. 

Among the purebreeds, Boxers (OR 7.2, 95% CI 4.5–11.6), American Pit Bull Terriers (OR 5.4, 95% CI 1.7–17.3), French Bulldogs (OR 4.4, 95% CI 1.6–12.2), and Labrador Retrievers (OR 2.6, 95% CI 1.5–4.5) were statistically overrepresented in the MCT-group compared with the VTH group. Dogs with MCTs were significantly older than in the VTH group (*p* < 0.0001). Among the MCT-group, there were more neutered dogs and spayed females than intact dogs and intact females compared to the VTH group, respectively (OR 2.1, 95% CI 1.4–3.2; OR 2.3, 95% CI 1.3–4.0) (Table 2).

The median body weight was 30.0 kg (range 5.0–50.0 kg). MCT sizes were available in 73 (74.5%) cases and the median size was 2.0 cm (range 0.5–10.0 cm). Eighty dogs (82%) presented with single nodules, while 18 had multiple nodules. Table 3 summarises the anatomical location, size, presence of single or multiple MCTs, ulceration, lymph node and distant metastasis, WHO stages, histopathological grading, surgical margins, and treatments. 

No associations were found between multiple or single MCTs and breed, age, sex, sexual status, body weight, size, cutaneous ulceration, anatomical location, lymph node status, distant metastasis, histological grade, and margins. Ulceration and lymph node metastasis occurred more frequently in larger MCTs (OR 5.2, 95% CI 1.5–18.5; OR 7.1, 95% CI 1.8–28.9). Both ulcerations and MCTs larger than 3 cm were highly associated with lymph node metastasis (OR 24.8, 95% CI 1.2–510.3).

At the end of the study, 54 dogs (55%) had died, 26 dogs were still alive and 18 were lost at the follow-up. Of those dogs that died, ten were euthanised or died due to MCTs, and four died due to other neoplasias (two hemangiosarcoma, one oral tumour, one heart tumour). Two dogs died with neurological disorders, one with *ab ingestis* pneumonia, one with congestive heart failure, and one with severe thrombocytopenia. In twenty-six cases, death seemed unrelated to MCTs. Nine dogs were euthanised due to unknown diseases. These latter thirty-four dogs were excluded from cause of death survival analysis. 

MCTs recurred in 14 out of 80 dogs (18%) and a de novo MCT in one dog. The recurrences were significantly associated with MCT-related death (OR 10.5, 95% CI 2.7–50.7, *p* = 0.0040). 

The median survival time (MST) was 509 days (range 8–2122 days) in deceased dogs. The median follow-up of living dogs was 1410 days (range 347–2576 days). No follow-up information was available for 18 dogs (18%), which were excluded from the statistical analysis. The MST was significantly shorter in dogs with MCT-related death (*n* = 10) compared with documented MCT-unrelated deceased dogs (*n* = 9) (MST 175.5 and 573 days, respectively; *p* = 0.0045). No associations were found between MST and breed, sex, sexual status, body weight, anatomical location, multinodular presentation, ulceration, distant metastasis, both Patnaik and Kiupel histological gradings, and histological margins. 

**Figure 1:**
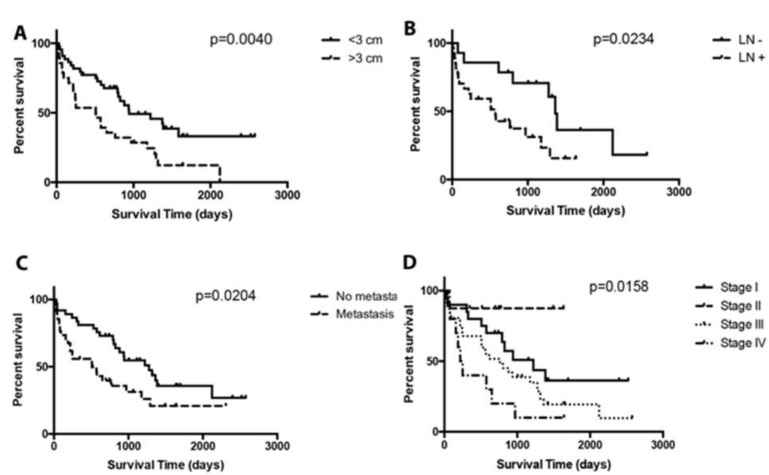
Kaplan–Meier survival plot of dogs with MCTs according to tumour size, presence of documented lymph node (LN) metastasis, presence of any type of metastasis (MTS) and WHO staging system classification. Vertical tick marks along survival curves represent censored dogs. (**A**) The median survival time for dogs with MCTs > 3 cm was 510 days and for dogs with MCTs < 3 cm, the median ST was 943 days (*p* = 0.0040). (**B**) The median survival time for dogs with lymph node metastasis (LN+) was 570 days, and for dogs without lymph node metastasis (LN−) it was 1362 days (*p* = 0.0234). (**C**) The median survival time for dogs with metastasis (MTS+) was 511 days, and for dogs without metastasis (MTS−) it was 1276 days (*p* = 0.0204). (**D**) The median survival times for dogs with stage I, stage II, stage III, and stage IV were 1221, not reached, 763, and 234 days, respectively (*p* = 0.0158).

Dogs with MCTs larger than 3 cm lived for shorter periods than dogs with smaller MCTs (MST 510 and 943 days, respectively; *p* = 0.0040) (Figure 1). Dogs with lymph node metastasis (*n* = 27) lived for shorter periods than dogs with presumed (*n* = 43) or documented (*n* = 14) absence of lymph node metastasis (*p* = 0.0492 and *p* = 0.0234, respectively). The presence of metastasis was associated with a shorter survival time (*p* = 0.0204, OR 2.5, 95% CI 1.400–4.453). WHO stages were associated with clinical outcome (*p* = 0.0158), although there were no differences between stages I and II, stages I and III, and stages III and IV. 

## 4. Discussion

In the present study, the Boxer, American Pit Bull Terrier, French Bulldog, and Labrador Retriever breeds were overrepresented in MCT dogs. The Boxer and Bulldog-related breeds, including Bullmastiffs, Boston Terriers, and Staffordshire Bull Terriers, have already been reported to be at increased risk of developing MCTs [6]. These breeds are considered phylogenetically close to each other [15]. The hypothesis postulated by Dr. Peters almost 50 years ago that the predisposition of these breeds to develop MCTs may be linked to a common ancestry, thus, seems more probable [16]. Moreover, the dogs referred to our VTH come from an area no further away than 100 km, thus suggesting a possible strict genealogical link between dogs belonging to the same breed. However, in the present study, no information regarding origin of different breeds was available, making any further analysis not feasible.

In line with the literature, in our study MCTs occurred in middle-old aged dogs, which were significantly older than the VTH group [7]. We found no association between histological grade and age, as previously reported [17]. However, the low rate of high-grade tumours in our population might have influenced the final results.

Neutered dogs and spayed females were significantly overrepresented compared to intact dogs and intact females, respectively. The influence of neutering in canine cancer is still not clear. In the last ten years, some studies have focused on the role of neutering in MCTs and other tumour developments. A study evaluating Golden Retrievers found a difference between the percentage of MCTs diagnosed in spayed females (2.3–5.7%) compared to intact ones (0%) [18]. However, the same authors failed to find similar results in Labrador Retrievers [19]. Other studies have suggested an increased risk of MCT development in modified sexual status, especially in spayed females, with an OR of up to 4.5 [20,21].

We found a strong association between MCTs larger than 3 cm and both ulceration and lymph node metastasis. More interestingly, ulcerated MCTs larger than 3 cm had an increased risk, of up to 24.8-fold, of showing lymph node metastasis. Recently, normal-sized regional lymph nodes in ninety-three dogs with cutaneous MCTs were evaluated histopathologically [22]. Lymph node metastases were found in almost two thirds of our cases, which was positively associated with tumours larger than 3 cm. Although we collected no specific information on regional lymph nodes, our data support, as previously reported, that MCTs larger than 3 cm tend to be associated with lymph node metastases. The excision of regional lymph nodes is thus crucial for correct staging. 

Size and lymph node metastases, as well as distant metastases, were associated with shorter survival times. Although lymph node metastasis has been reported to be associated with more aggressive disease and worse outcomes, some studies have suggested the role of surgery, including lymphadenectomy, and/or adjuvant therapies in improving survival rates [3,8,23]. 

However, the development of new staging strategies, such as lymph node mapping, has shown how difficult it is to recognise the sentinel lymph node, which seems to be different from the regional lymph node in more than 50% of canine MCTs [24]. Moreover, when MCTs occur on the trunk, it is not easy to establish which and where the regional lymph node is, making nodal excision challenging.

Unfortunately, the present study has no information on the lymph node size and type of treatment applied in dogs with nodal metastasis, which highlights a bias in the survival interpretation. However, as stated above, the high prevalence of nodal metastasis in normal-sized lymph nodes [22], together with the previously reported improved outcome with lymphadenectomy or other adjuvant therapies [3,8,23], mean that the regional/sentinel lymph node plays a key role in the staging and treatment of canine MCTs. 

The present study has some limitations. Firstly, the retrospective nature did not allow the authors to collect complete clinical and survival information. Secondly, during the six-year study period, different clinicians managed the 98 dogs with MCTs, meaning the choice of treatment was not standardised. Moreover, this period overlapped with the incoming of the tyrosine-kinase inhibitors era, which gave clinicians a first-line drug treatment for unresectable MCTs. The lack of treatment standardization may have affected the survival data. Lastly, follow-up information was collected via phone call with the owners in some cases, making recall bias possible and results might not be accurate 

## 5. Conclusions

In the present study, breed, sex, and spay/neuter status were associated with increased risk for MCT occurrence. Both MCTs larger than 3 cm and ulceration were positively associated with lymph node metastasis at presentation. Even if the presence of metastasis, recurrences, and MCT-related death were associated with shorter survival, these results should be interpreted with caution given possible follow-up collection biases.

## Figures and Tables

**Table 1:** Odds Ratios (OR) and 95% confidence interval for MCT frequency by breed.

Breed	MCT Dogs (%)	VTH Dogs (%)	*p*-Value	OR (95% CI)
Mixed †	24 (24.5)	3576 (27.1)	0.64	
Boxer †	23 (23.5)	537 (4.1)	<0.0001	7.2 (4.5–11.6)
Labrador Ret. †	15 (15.3)	866 (6.6)	0.0011	2.6 (1.5–4.5)
Golden Ret. †	7 (7.1)	430 (3.3)	0.06	
French Bulldog ‡	4 (4.1)	126 (1.0)	0.0156	4.4 (1.6–12.2)
American Pit Bull Terrier ‡	3 (3.1)	77 (0.6)	0.0213	5.3 (1.7–17.3)
English Setter ‡	3 (3.1)	240 (1.8)	0.27	
Bolognese ‡	2 (2.0)	79 (0.6)	0.12	
Pug ‡	2 (2.0)	135 (1.0)	0.27	
Miniature Poodle ‡	1 (1.0)	450 (3.4)	0.27	
Dachshund ‡	1 (1.0)	391 (3.0)	0.37	
Dobermann Pinscher ‡	1 (1.0)	186 (1.4)	1.00	
German Shepherd ‡	1 (1.0)	743 (5.6)	0.045	0.2 (0.0–1.3)
Pointer ‡	1 (1.0)	61 (0.5)	0.37	
Shih tzu ‡	1 (1.0)	127 (1.0)	0.61	
Springer Spaniel ‡	1 (1.0)	159 (1.2)	1.00	
Yorkshire Terrier ‡	1 (1.0)	180 (1.4)	1.00	

MCT: Mast cell tumour group; VTH: Veterinary teaching hospital caselog; OR: Odds Ratio. Breeds were analysed using the Chi-square (†) or the Fisher’s exact (‡) test.

**Table 2:** Odds Ratios (OR) and 95% confidence interval for MCT frequency by sex and sexual status.

Sex and Spay/Neuter Status	MCT Dogs (%)	VTH Dogs (%)	*p*-Value	OR (95% CI)
Sex				
Male	47 (48.0)	6739 (51.5)	0.48 †	
Female	51 (52.0)	6338 (48.5)		
Neutered male	4 (4.1)	312 (2.4)	0.28 ‡	
Intact male	43 (43.9)	6427 (49.1)		
Intact female	25 (25.5)	4372 (33.4)	0.0022 †	1
Spayed female	26 (26.5)	1966 (15.1)		2.31 (1.33–4.02)
Sexual status				
Intact	68 (69.4)	10,799 (82.6)	0.0006 †	1
Spay/neuter	30 (30.6)	2278 (17.4)		2.09 (1.36–3.22)

MCT: Mast cell tumour group; VTH: veterinary teaching hospital caselog; OR: Odds Ratio. Sex and sexual status were analysed using the Chi-square (†) or the Fisher’s exact (‡) test.

**Table 3:** Clinical data distribution regarding the ninety-eight dogs with MCT.

Variable	Dogs *n* (%)
Localisation † (*n* = 90)	
Extremities	15 (15.3)
Trunk	42 (42.8)
Head	16 (16.3)
Miscellaneous	17 (17.3)
Single/Multiple (*n* = 98)	
Single	80 (81.6)
Multiple	18 (18.4)
Size ‡ (*n* = 86)	
<3 cm	53 (60.2)
>3 cm	33 (39.8)
Ulceration (*n* = 89)	
Present	15 (16.8)
Absent	74 (83.1)
Presence of metastasis	
Lymph node (*n* = 48)	28 (58.3)
Spleen and/or liver (*n* = 88)	12 (13.6)
WHO clinical staging (*n* = 84)	
0	1 (1.2)
I	25 (29.8)
II	8 (9.5)
III	38 (45.2)
IV	12 (14.3)
Histological grading § (*n* = 68)	
G1P	8 (11.8)
G2P	54 (79.4)
G3P	2 (2.9)
G1P-LGK	2 (6.0)
G2P-LGK	27 (81.8)
G2P-HGK	1 (3.0)
G3P-HGK	3 (9.1)
Subcutaneous	4 (5.9)
Histological margins (*n* = 53)	
Complete	30 (56.7)
Narrow	10 (18.9)
Incomplete	13 (24.5)
Treatment (*n* = 82)	
Surgery	40 (48.8)
Chemotherapy and/or TKI	8 (9.8)
Surgery and radiotherapy	2 (2.4)
Surgery and chemotherapy and/or TKI	22 (26.8)
Radiotherapy and chemotherapy and/or TKI	2 (2.4)
No therapy	12 (14.6)

G*n*P: Patnaik grading (*n* ranged from 1 to 3); HGK: high grade Kiupel; LGK: low grade Kiupel; TKI: tyrosine-kinase inhibitors. † Eight dogs presented multiple MCT with at least two different localisations; ‡ Median size 2.0 cm (range 1.0–6.0 cm) and 2.3 cm (range 0.5–10.0 cm) in multiple and single MCT, respectively.
